# Fezolinetant’s efficacy and safety in treatment of vasomotor symptoms in postmenopausal women: a meta-analysis and GRADE evaluation of randomized controlled trials

**DOI:** 10.1186/s40001-025-02279-y

**Published:** 2025-01-23

**Authors:** Abdallah R. Allam, Mohamed Salah Alhateem, Abdelrahman Mohamed Mahmoud

**Affiliations:** Faculty of Medicine, Menoufia University, Yassin Abdelghaffar Street From Gamal Abdelnaser Street, Shebin Al-Kom, 32511 Menoufia Egypt

**Keywords:** Vasomotor symptoms, Fezolinetant, Postmenopausal, Meta-analysis

## Abstract

**Background:**

Postmenopausal women are more likely to experience vasomotor symptoms (VMS), such as heat sensation and sweating. Recent trials have investigated fezolinetant in the treatment of VMS in postmenopausal women. Our study aims to conduct a meta-analysis of these trials in order to estimate fezolinetant’s effectiveness and safety in the management of VMS in postmenopausal women.

**Method:**

We searched Cochrane, PubMed, Scopus, and Web of Science for all published randomized controlled trials. Review Manager Software was used for the meta-analysis. The quality of evidence was graded using the Grading of Recommendations, Assessment, Development, and Evaluations (GRADE) framework.

**Results:**

Our study contained five trials with 3295 individuals with a mean age of 54.4 years. The frequency of VMS was significantly lower in the fezolinetant group compared to the placebo group [MD = − 2.42, 95% CI (− 2.81, − 2.04), *P* < 0.00001]. Additionally, when compared to the placebo group, the severity of VMS was significantly lower in the fezolinetant group [SMD = − 0.36, 95% CI (− 0.46, − 0.26), *P* < 0.00001]. Furthermore, there was no significant difference in the incidence of treatment-emergent adverse events (TEAEs) between the fezolinetant group and the placebo group [RR = 1.02, 95% CI (0.97, 1.07), *P* = 0.51].

**Conclusion:**

Fezolinetant is efficient and well-tolerated in the treatment of postmenopausal women with VMS.

**Supplementary Information:**

The online version contains supplementary material available at 10.1186/s40001-025-02279-y.

## Introduction

Up to 80% of women in the United States report experiencing vasomotor symptoms (VMS) during the menopausal transition [1], which last for a median of 7.4 years [2]. The majority of women classify VMS as moderate-to-severe [3], characterized by heat sensation and sweating that may force a halt to routine activities [4]. VMS can significantly reduce quality of life by causing physical and psychosocial impairment, which can have an influence on daily activities, social interactions, and work performance [5]. Additionally, the discomfort brought by VMS can negatively impact sleep quality [6]. Furthermore, anxiety and depression are also linked to VMS [7].

Hormone therapy (HT) is an effective treatment currently available for menopause-related VMS [8]. However, HT has been linked to common adverse effects (AEs) including breakthrough bleeding, breast tenderness, nausea, bloating, and mood fluctuations as well as an elevated risk of stroke and venous thromboembolism [9, 10]. HT is acknowledged in worldwide clinical practice recommendations, particularly for symptomatic women under the age of 60 or within 10 years of menopause. However, safety and tolerability issues have deterred VMS patients from using HT [8, 11]. As a result, women who suffer from VMS mainly and are unable or unwilling to take HT should seek out safe, effective, tailored nonhormonal therapy for relief.

Fezolinetant, a nonhormonal selective neurokinin-3 receptor (NK3R) antagonist, has emerged as a promising therapeutic option for the management of vasomotor symptoms (VMS) in postmenopausal women [12].

The efficacy of fezolinetant is rooted in its specific interaction with the neurokinin B (NKB)/NK3R pathway within the hypothalamus responsible for regulating the body’s temperature. During the menopausal transition, declining estrogen levels lead to a disruption in the normal regulatory functions of the hypothalamus, specifically affecting the kisspeptin/neurokinin B/dynorphin (KNDy) neurons. By selectively blocking NK3R, fezolinetant effectively reduces the activity of KNDy neurons, thereby alleviating the frequency and severity of VMS [13–15].

In May 2023, fezolinetant received approval from the United States Food and Drug Administration (FDA) for the treatment of VMS associated with menopause, marking a significant milestone in menopausal therapeutics. Recent studies have investigated fezolinetant a potential treatment for VMS in postmenopausal women. We conducted a systematic review and meta-analysis of the available RCTs to evaluate the efficacy and tolerability of fezolinetant in the treatment of VMS in postmenopausal women.

## Methods

In order to perform this study, we followed the “Preferred reporting items for systematic review and meta-analysis” (PRISMA) declaration [16]. In addition, we followed the guidelines for a systematic review of interventions that were reported in the Cochran Handbook [17]. In order to evaluate the quality of this study, we also used the Grading of Recommendations Assessment, Development, and Evaluation tool [18].

### Literature search

We conducted a comprehensive searching for all published RCTs till March 2023 through PubMed, Scopus, Web of Science, and Cochrane using the following search terms: “Fezolinetant”, “ESN364”, “Menopause”, “Change of Life, Female”.

### Inclusion and exclusion criteria

We enrolled postmenopausal females having vasomotor symptoms in randomized controlled trials that compared Fezolinetant with the placebo and reported on the drug’s efficacy or safety outcomes. In vitro research, overlapping datasets, book chapters, thesis, reviews, editorials, abstract-only papers at conferences, non-English articles, cohort studies, and case–control studies were excluded from our study.

### Study selection and data extraction

In order to remove duplicated studies from the review, we used the systematic review accelerator tool [19]. Next, we screened the titles and abstracts of the included studies, and then the eligible studies were subjected to full-text screening prior to their inclusion in the final analysis. A predefined data extraction sheet was used to extract the data. The data extracted included a summary of each study that was included, the baseline demographics for the study population, and the safety and efficacy of the included studies.

### Outcomes

The frequency and severity of VMS, the Patient Global Impression of Change in Sleep Disturbance (PGI-C SD), the Patient Global Impression of Severity in Sleep Disturbance (PGI-S SD), the Menopause-Specific Quality of Life (MENQOL), and the Patient-Reported Outcomes Measurement Information System Sleep Disturbance Short Form 8b (PROMIS SD SF 8b) were among the efficacy outcomes. Treatment Emergent Adverse Events (TEAEs), treatment Related AEs, Serious TEAEs, and TEAEs leading to treatment discontinuation were among the safety outcomes.

### Risk of bias

Our assessment of the potential for bias in the included studies was based on the Cochrane Risk of Bias Assessment Tool 2 (ROB-2) [20]. This version of ROB looked at the randomization process, deviations from the intended interventions, missing outcome data, the measurement of outcomes, the selection of reported outcomes, and overall bias risks. In each domain, we classified it as either low, high, or some concerns.

### Data synthesis

For this meta-analysis, we used Review Manager Software (Revman 5.4). Data for Fezolinetant and a placebo were compared. The administration of the Fezolinetant dosage and timing were taken into consideration during the sub-group analysis. A 95% confidence interval (CI) and a mean difference (MD) or standardized mean difference (SMD) were used to analyze the continuous data using the inverse variance technique. A Mantel–Haenszel analysis was performed on the dichotomous data, employing a risk ratio (RR) and a 95% CI. At a *P*-value < 0.05, a difference was deemed statistically significant. When the Chi-square *P* < 0.1 and *I*-square test (*I*^2^) > 50%, the data were deemed heterogeneous [21]. A leave-one-out sensitivity analysis and a random-effect models were used if the data were heterogeneous. A fixed-effects model was applied otherwise.

### Certainty of evidence

Two independent reviewers (A.R.A., A.M.M.) evaluated the certainty of the evidence for the outcome of hospitalization using the GRADE (Grading of Recommendations, Assessment, Development, and Evaluations) framework.

## Results

### Literature search results

**Fig. 1:**
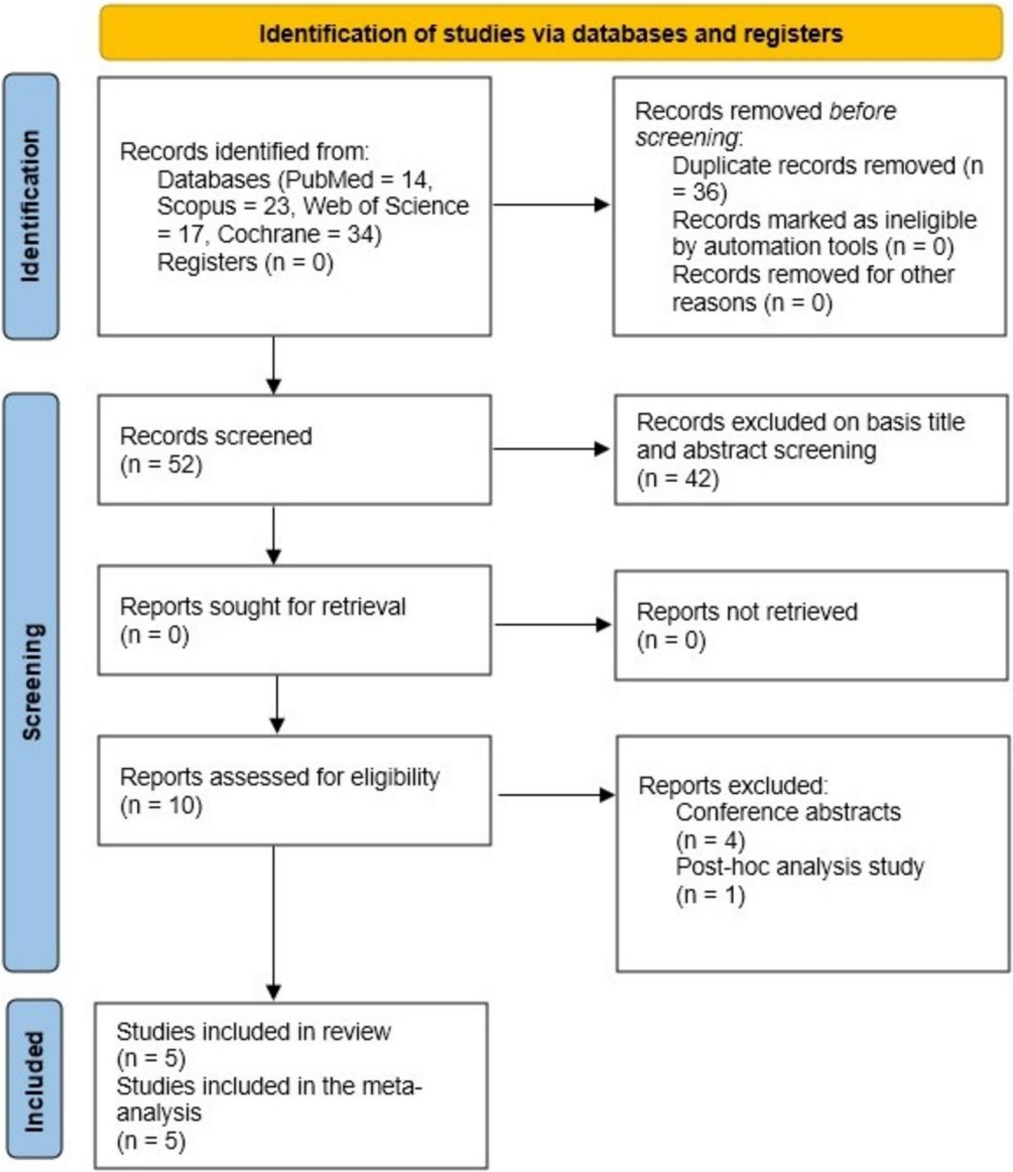
PRISMA flow diagram

After removing duplicate studies, the search yielded 52 studies instead of the original 88. Out of the 52 studies total, only 10 were eligible for full-text screening, and 5 [12, 22–25] of those studies were used in this analysis (Fig. 1).

### Characteristics of included studies

There were 3295 participants in this study with an average body mass index of 28.05 kg/m^2^ and a mean age of 54.4 years. Fezolinetant was administered to participants in the following doses: 30 mg once daily for 994, 45 mg once daily for 949, 60 mg once daily for 45, 120 mg once daily for 44, 15 mg twice daily for 45, 30 mg twice daily for 43, 60 mg twice daily for 45, 90 mg twice daily for 87; and placebo for 1039. Tables 1 and 2 present, respectively, summaries of the included studies and participant baseline characteristics.

**Table 1:** Summary of included studies

ID	Design	Dose	Type of administration	Duration of treatment	Participants	NCT	Site	Inclusion criteria	Exclusion criteria	Conclusion
Lederman 2023	Phase 3	30, 45 mg once daily	Oral	12 weeks	522	NCT04003155	USA, Canada, Czech Republic, Hungary, Poland, Spain, and UK	The inclusion criteria included being a female, 40 to 65 years old at screening, having a BMI of 18 to 38 kg/m^2^, seeking treatment or relief for menopause-related VMS, having spontaneous amenorrhea for at least 12 months prior to screening, and having experienced at least seven to eight moderate-to-severe hot flashes within the 10 days prior to randomization	Receiving HRT, hormonal contraceptives, or any other form of treatment for VMS, as well as a prior or present history of a malignant tumor, with the exception of basal cell carcinoma, were among the exclusion criteria	According to the findings of SKYLIGHT 1, fezolinetant 30 mg and 45 mg once daily were effective for the long-term management of mild to severe VMS related to menopause. VMS dramatically decreased during the first week of therapy, remained stable until week 12, and continued for 52 weeks without showing signs of tachyphylaxis. For many women who experience vasomotor symptoms, NK3R antagonists have the potential to offer alternative nonhormonal therapy alternatives that fulfill unmet needs
Johnson 2023	Phase 3	30, 45 mg once daily	Oral	12 weeks	500	NCT04003142	USA, Canada, Czechia, Latvia, Poland, Spain, and UK	The inclusion criteria were being a woman at birth, being between the ages of 40 and 65 at screening, having a BMI between 18 and 38 kg/m^2^, seeking therapy or relief for menopause-related VMS both during the screening visit and at the time of the visit, and having spontaneous amenorrhea for at least 12 months	Taking HRT, hormonal contraceptives, or receiving any treatment for VMS, as well as having a past or present history of a malignant tumor other than basal cell carcinoma, were among the exclusion criteria	For the treatment of moderate-to-severe VMS related to menopause, fezolinetant 30 and 45 mg once day showed efficacy and were well tolerated. The fezolinetant groups experienced a daily reduction of 2 to 3 VMS episodes greater than the placebo from baseline to week 12 with a rapid start of impact by week 1, a full effect by week 4, and a maintained effect through 52 weeks. Fezolinetant 45 mg also markedly enhanced patient-reported sleep quality. These results encourage the further research and development of fezolinetant as a novel nonhormonal therapy option for menopausal-related VMS
Neal-Perry 2023	Phase 3	30, 45 mg once daily	Oral	52 weeks	1830	NCT04003389	–	Participants had a BMI between 18 and 38, inclusive, and were proven to be postmenopausal by experiencing spontaneous amenorrhea for 12 months or more. Participants ranged in age from 40 to 65 and were seeking therapy for VMS associated with menopause	Participation was not permitted if the patient was taking strong or moderate cytochrome P450 1A2 inhibitors, HRT, hormonal contraceptives, or any other prescription, over-the-counter, or herbal treatment for VMS, unless the patient had drug-specific washout periods based on the known half-lives of the drugs	Fezolinetant’s effectiveness in lowering the frequency and severity of VMS was shown by phase 3 results from SKYLIGHT 1 and 2, which also revealed the drug’s safety profile when compared to a placebo over a 12-week period. Results from SKYLIGHT 4 give more proof that fezolinetant is safe throughout the course of a 52-week treatment period, and the data support the drug’s ongoing development as a novel, nonhormonal therapy option for moderate-to-severe VMS linked to menopause
Fraser 2020	Phase 2	30, 60, 120 mg once daily. 15, 30, 60, 90 twice daily	Oral	12 weeks	356	NCT03192176	USA	Postmenopausal women between the ages of 40 and 65 who experienced 50 moderate or severe VMS episodes per week, as determined by seven consecutive days of VMS recordings from any point throughout the 35-day screening period, qualified as participants	Malignant tumors (other than basal cell carcinoma), endometrial hyperplasia or uterine/endometrial cancer, unexplained uterine bleeding, seizures or other convulsive disorders, severe allergies, or intolerances to drugs generally or any of the excipients in the study medication, drug, or alcohol abuse, or any of these conditions were considered grounds for exclusion from the study	According to the study’s findings, fezolinetant is a nonhormone medication that quickly reduces moderate-to-severe menopausal-related VMS and is well tolerated. Effectiveness was shown at various dose levels and with once- and twice-daily administration, and with some doses, efficacy was seen within the first week. It is necessary to conduct larger and longer phase 3 trials on women with VMS related to menopause in order to assess the efficacy and safety profile of fezolinetant more thoroughly
Depypere 2019	Phase 2	90 mg twice daily	Oral	12 weeks	87	EudraCT2015-002578-20	Belgium	Women between the ages of 40 and 65 who had achieved menopause and were dealing with mild to severe VMS were enrolled in this study. Menopause was deemed to begin after twelve straight months of spontaneous amenorrhea	Any medical condition that could skew results, such as a history of drug or alcohol misuse, a suicide attempt within the past three years, active liver disease or jaundice, high liver function enzymes, or impaired kidney function, led to the exclusion of subjects from the study	The incidence and severity of moderate/severe VMS were greatly reduced by fezolinetant, and this effect was visible as early as the first day of treatment. The efficacy of fezolinetant was good, and it had no impact on E2 levels. The study’s findings regarding fezolinetant’s efficacy and safety indicate its prospective usage as a nonhormonal therapeutic option for menopausal women with VMS

*BMI* body mass index, *VMS* vasomotor symptoms, *HRT* hormonal replacement therapy, *NK3R* nonhormonal selective neurokinin-3 receptor, *E2* estradiol

**Table 2:** Baseline characteristics of included participants

ID	Dose	Age (years), *M* (SD)	BMI (kg/m^2^), *M* (SD)	Race (white), *N* (%)	Noncurrent smoker, *N* (%)	Time since onset of VMS (months), *M* (SD)	Amenorrhea (yes), *N* (%)	Hysterectomy (no), *N* (%)	Oophorectomy (no), *N* (%)
Lederman 2023	30 mg once daily	54.2 (4.9)	28·14 (4·83)	148 (86%)	152 (87%)	77·4 (66.3)	170 (98%)	113 (65%)	137 (79%)
	45 mg once daily	54.2 (5.1)	28·28 (4·35)	141 (82%)	151 (87%)	71·9 (59.3)	171 (99%)	117 (68%)	136 (79%)
	Placebo	54.7 (4.8)	28·19 (4·28)	142 (81%)	153 (87%)	81·9 (73.6)	170 (97%)	124 (71%)	137 (78%)
Johnson 2023	30 mg once daily	53.9 (4.9)	27.94 (3.25)	131 (78.9%)	132 (79.5%)	76.2 (61.16)	163 (98.2%)	113 (68.1%)	132 (79.5%)
	45 mg once daily	54.3 (5.4)	27.91 (3.25)	132 (79.0%)	133 (79.6%)	81.7 (65.67)	162 (97.0%)	111 (66.5%)	129 (77.2%)
	Placebo	54.7 (4.6)	28.6 (3.12)	134 (80.2%)	132 (79%)	81.9 (60.16)	159 (95.2%)	116 (69.5%)	130 (77.8%)
Neal-Perry 2023	30 mg once daily	54.7 (4.7)	28.46 (4.5)	479 (78.5%)	495 (81%)	–	–	511 (83.6%)	536 (87.7%)
	45 mg once daily	54.7 (4.8)	28.46 (4.7)	479 (78.8%)	493 (81%)	–	–	495 (81.3%)	523 (85.9%)
	Placebo	54.9 (4.8)	28.26 (4.6)	502 (82.3%)	493 (80.2%)	–	–	483 (79.2%)	524 (85.9%)
Fraser 2020	15 mg twice daily	53.7 (5.0)	29.3 (4.3)	37 (82.2%)	35 (77.8%)	–	–	–	–
	30 mg twice daily	53.9 (3.8)	28.3 (4.0)	31 (72.1%)	38 (88.4%)	–	–	–	–
	60 mg twice daily	54.6 (5.0)	29.1 (5.2)	28 (62.2%)	37 (82.2%)	–	–	–	–
	90 mg twice daily	54.9 (4.0)	27.3 (4.6)	36 (81.8%)	40 (90.9%)	–	–	–	–
	30 mg four times daily	52.7 (3.8)	28.8 (4.0)	31 (72.1%)	40 (93%)	–	–	–	–
	60 mg four times daily	55.0 (4.9)	28.3 (4.4)	34 (75.6%)	34 (75.5%)	–	–	–	–
	120 mg four times daily	56.8 (4.4)	28.8 (4.9)	30 (68.2%)	41 (93.2%)	–	–	–	–
	Placebo	54.8 (5.5)	27.3 (4.8)	30 (69.8%)	40 (93%)	–	–	–	–
Depypere 2019	90 mg twice daily	53.3 (4.03)	25.1 (4.71)	42 (97.7%)	–	–	–	–	–
	Placebo	53.7 (4.25)	26.5 (6.15)	44 (100%)	–	–	–	–	–

*BMI* body mass index, *M* mean, *SD* standard deviation, *N* number, *VMS* vasomotor symptoms

### Risk-of-bias results

**Fig. 2:**
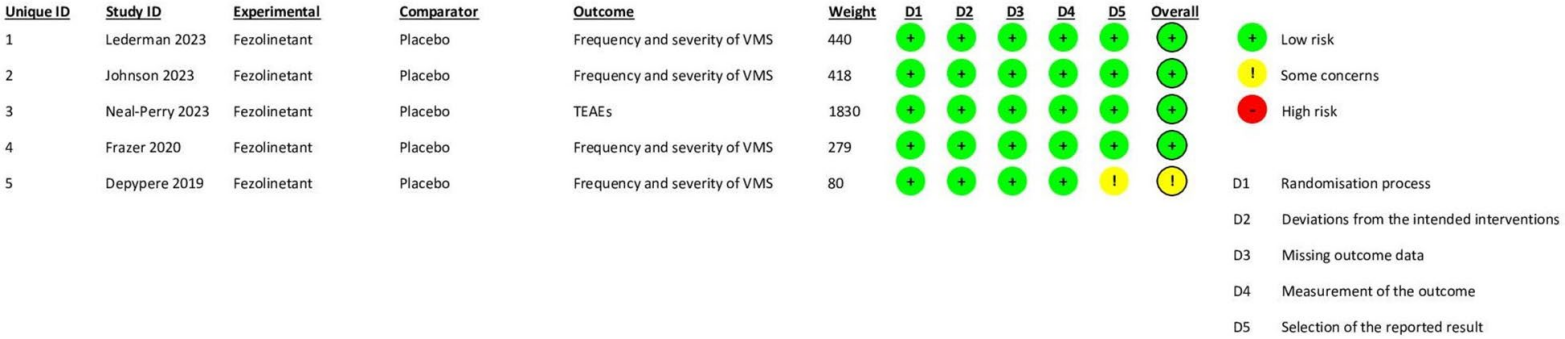
Risk-of-bias graph

All studies had a low risk of bias, except for Depypere et al. [22] which raised some concerns about the selection of presented outcomes as well as the overall risk-of-bias domains. Figure 2 and Supplementary Fig. 1, respectively, illustrate a risk-of-bias graph and a risk-of-bias summary.

### Efficacy outcomes

#### Frequency of VMS

**Fig. 3:**
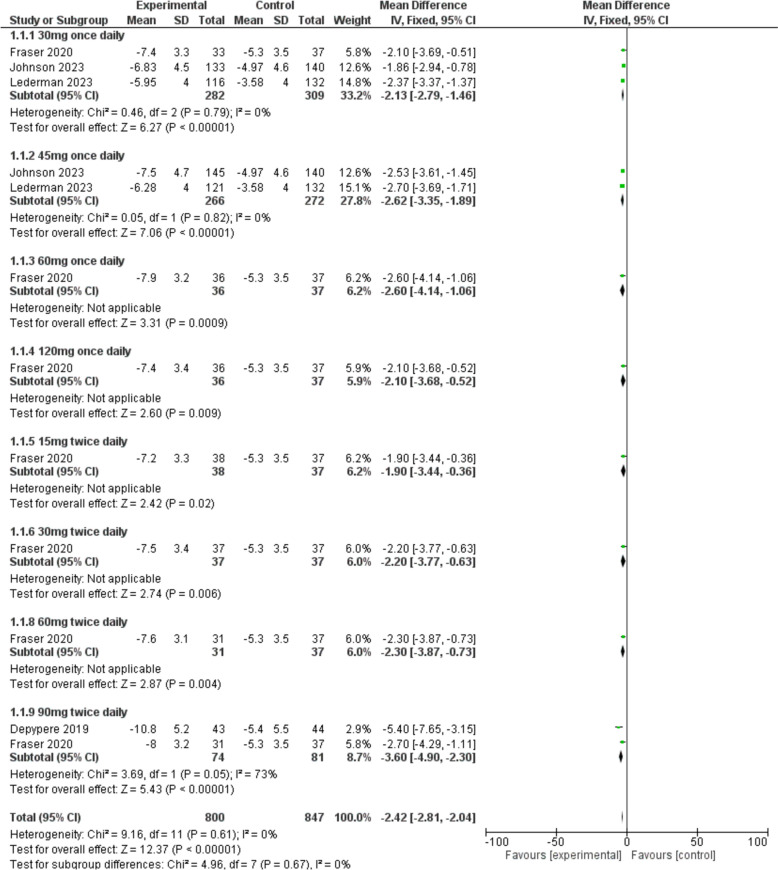
A forest plot comparing the frequency of VMS in the fezolinetant group versus the placebo group

The frequency of VMS was significantly reduced in the fezolinetant group compared to the placebo group [MD = − 2.42, 95% CI (− 2.81, − 2.04), *P* < 0.00001]. The sub-group analysis also demonstrated that fezolinetant at both doses of 30 mg [MD = − 2.13, 95% CI (− 2.79, − 1.46), *P* < 0.00001] and 45 mg [MD = − 2.62, 95% CI (− 3.35, − 1.89), *P* < 0.00001] once daily was significantly superior to placebo (Fig. 3).

#### Severity of VMS

**Fig. 4:**
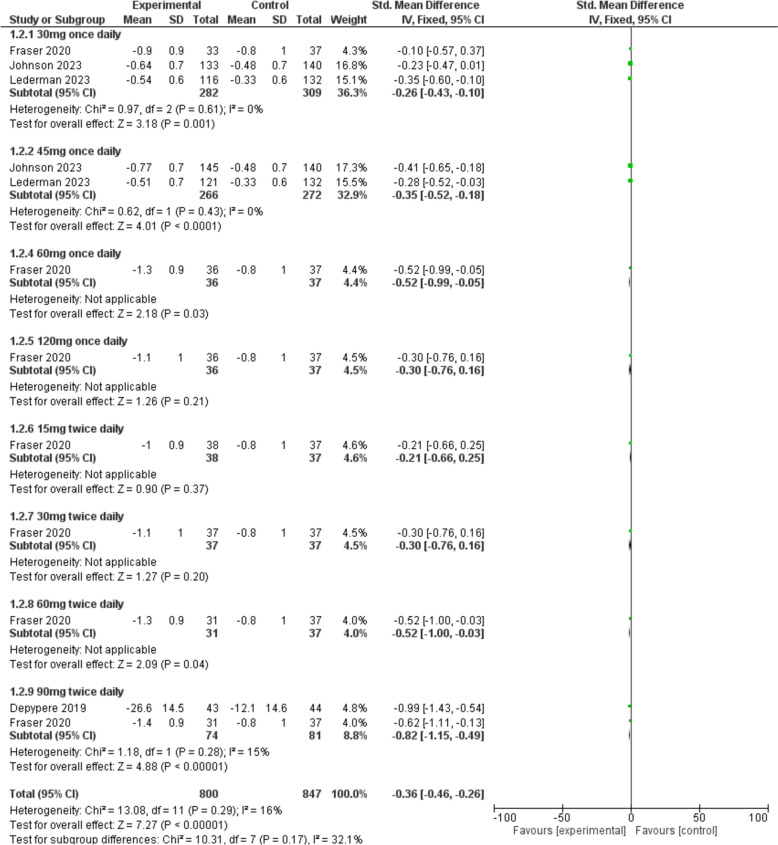
A forest plot comparing the severity of VMS in the fezolinetant group versus the placebo group

The severity of VMS was significantly reduced in the fezolinetant group compared to the placebo group [SMD = − 0.36, 95% CI (− 0.46, − 0.26), *P* < 0.00001]. The sub-group analysis also demonstrated that fezolinetant at both doses of 30 mg [SMD = − 0.26, 95% CI (− 0.43, − 0.10), *P* = 0.001] and 45 mg [SMD = − 0.35, 95% CI (− 0.52, − 0.18), *P* < 0.0001] once daily was significantly superior to placebo (Fig. 4).

#### PROMIS SD SF 8b

**Fig. 5:**
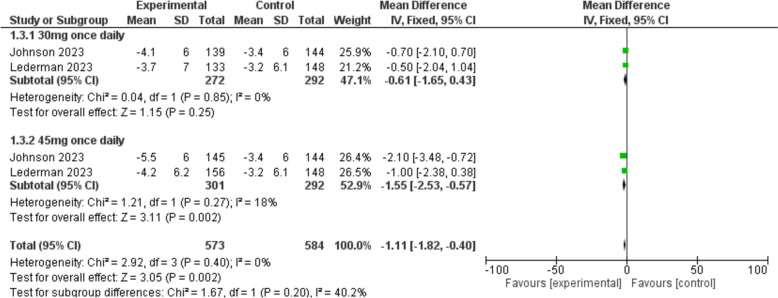
A forest plot comparing the total score of PROMIS SD SF 8b in the fezolinetant group versus the placebo group

This outcome was reported in two studies [23, 24]. The score of PROMIS SD SF 8b was significantly reduced in the fezolinetant group compared to the placebo group [MD = − 1.11, 95% CI (− 1.82, − 0.40), *P* = 0.002]. The sub-group analysis also demonstrated that fezolinetant 45 mg once daily was significantly superior to placebo [MD = − 1.55, 95% CI (− 2.53, − 0.57), *P* = 0.002] (Fig. 5).

#### MENQOL

**Fig. 6:**
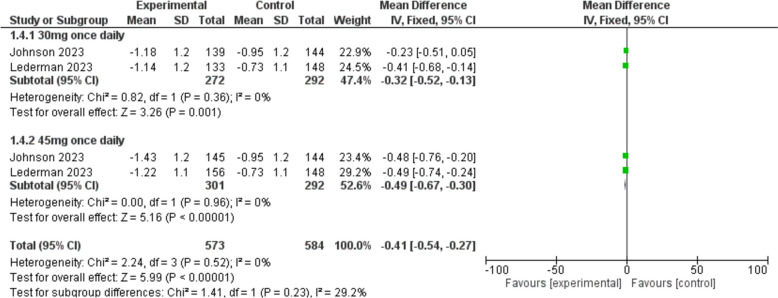
A forest plot comparing the score of MENQOL in the fezolinetant group versus the placebo group

This outcome was reported in two studies [23, 24]. The MENQOL score was significantly reduced in the fezolinetant group compared to the placebo group [MD = − 0.41, 95% CI (− 0.54, − 0.27), *P* < 0.00001]. The sub-group analysis also demonstrated that fezolinetant at both doses of 30 mg [MD = − 0.32, 95% CI (− 0.52, − 0.13), *P* = 0.001] and 45 mg [MD = − 0.49, 95% CI (− 0.67, − 0.30), *P* < 0.00001] once daily was significantly superior to placebo (Fig. 6).

#### PGI-C SD

This outcome was reported in two studies [23, 24]. Compared to the placebo group, the fezolinetant group had a significantly higher incidence of much better outcome [RR = 1.63, 95% CI (1.30, 2.04), *P* < 0.0001] (Figure S2 A). There was no significant difference between the fezolinetant group and the placebo group in terms of moderately better outcome [RR = 1.15, 95% CI (0.92, 1.44), *P* = 0.22] (Figure S2 B). In addition, there was no significant difference between the fezolinetant group and the placebo group in terms of a little better outcome [RR = 1.04, 95% CI (0.87, 1.26), *P* = 0.65] (Figure S2 C). Compared to the placebo group, the fezolinetant group had a significantly lower incidence of no change outcome [RR = 0.69, 95% CI (0.56, 0.84), *P* = 0.0002] (Figure S2 D). Additionally, the fezolinetant group had a significantly lower incidence of a little worse outcome [RR = 0.46, 95% CI (0.27, 0.78), *P* = 0.004] (Figure S2 E). However, there was no significant difference between the fezolinetant group and the placebo group in terms of moderately worse outcome [RR = 0.97, 95% CI (0.54, 1.74), *P* = 0.92] (Figure S2 F).

There was no significant difference between the fezolinetant group and the placebo group in terms of much worse outcome [RR = 0.56, 95% CI (0.19, 1.67), *P* = 0.30] (Figure S2 G).

#### PGI-S SD

This outcome was reported in two studies [23, 24]. There was no significant difference between the fezolinetant group and the placebo group in terms of no problem outcome [RR = 1.16, 95% CI (0.92, 1.46), *P* = 0.20] (Figure S3 A). Additionally, there was no significant difference between the fezolinetant group and the placebo group in terms of mild problems outcome [RR = 1.07, 95% CI (0.94, 1.23), *P* = 0.31] (Figure S3 B). Furthermore, there was no significant difference between the fezolinetant group and the placebo group in terms of moderate problems outcome [RR = 1.07, 95% CI (0.91, 1.26), *P* = 0.39] (Figure S3 C). However, compared to the placebo group, the fezolinetant group had a significantly lower incidence of severe problems outcome [RR = 0.41, 95% CI (0.28, 0.60), *P* < 0.00001] (Figure S3 D).

### Safety outcomes

#### TEAEs

**Fig. 7:**
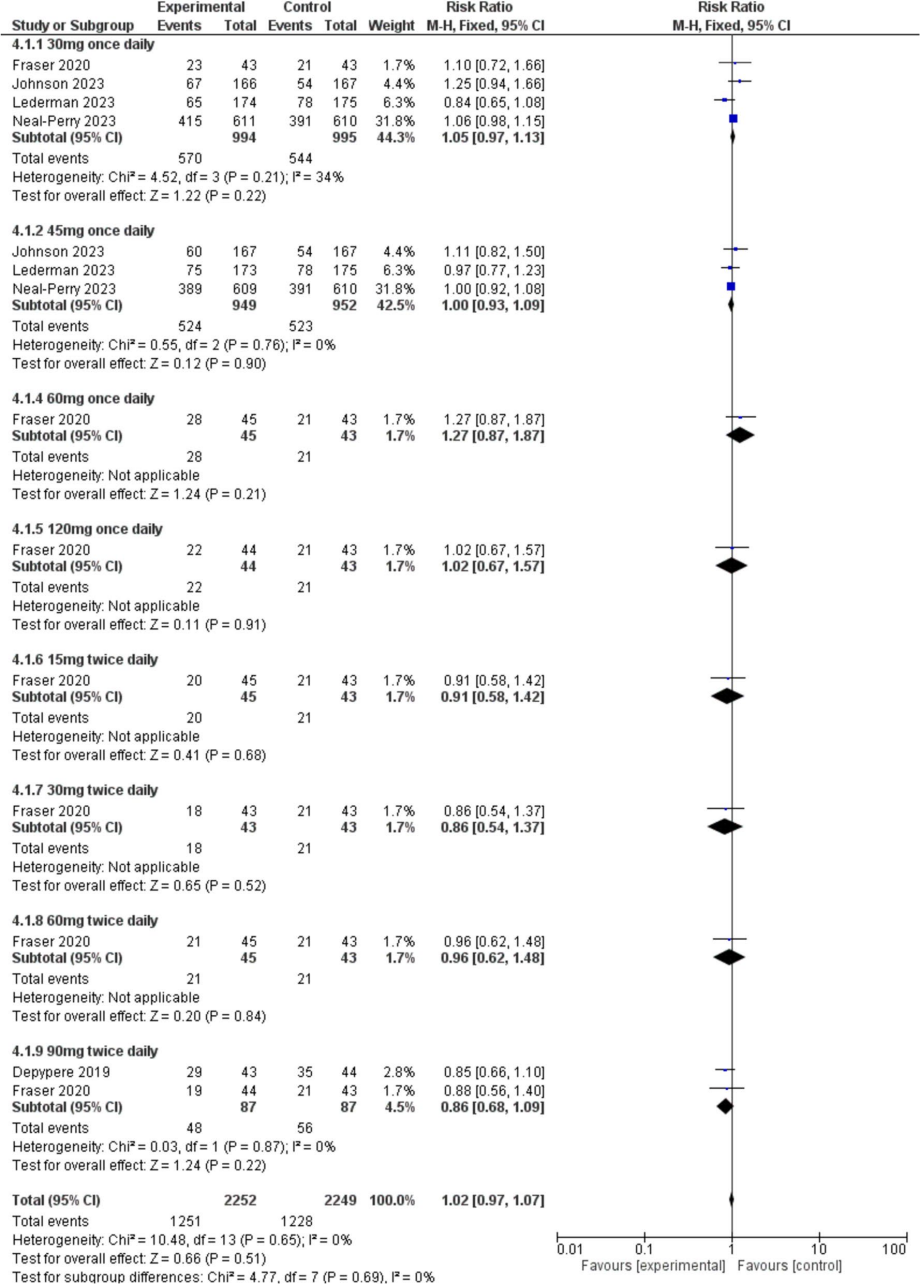
A forest plot comparing the prevalence of TEAEs in the fezolinetant group versus the placebo group

There was no significant difference between the fezolinetant group and the placebo group in terms of TEAEs [RR = 1.02, 95% CI (0.97, 1.07), *P* = 0.51] (Fig. 7).

#### Drug-related AEs

Compared to the placebo group, the fezolinetant group had a significantly higher incidence of drug-related AEs [RR = 1.50, 95% CI (1.32, 1.72), *P* < 0.00001]. However, the overall data were heterogeneous (*I*2 = 80%, *P* < 0.000001). In the sub-group analysis, placebo performed significantly better than fezolinetant 30 mg once daily [RR = 1.36, 95% CI (1.12, 1.66), *P* < 0.00001] (Figure S4 A). The data, however, were heterogeneous [*I*^2^ = 89%, *P* < 0.00001]. We eliminated Neal-Perry et al. [25] to address this heterogeneity and the results of sensitivity analysis showed a similar overall trend [RR = 2.76, 95% CI (1.95, 3.93), *P* < 0.00001] (Figure S4 B). Furthermore, placebo performed significantly better than fezolinetant 45 mg once daily [RR = 1.52, 95% CI (1.25, 1.84), *P* < 0.00001]. The data, however, were heterogeneous [*I*^2^ = 92%, *P* < 0.00001]. Eliminated Neal-Perry et al. [25] was removed to address this heterogeneity (Figure S4 C). The results after sensitivity analysis showed a similar overall trend [RR = 3.06, 95% CI (2.13, 4.37), *P* < 0.00001].

#### Serious TEAEs

Compared to the placebo group, the fezolinetant group had a significantly higher incidence of serious TEAEs [RR = 1.65, 95% CI (1.07, 2.54), *P* = 0.02] (Figure S5).

#### TEAEs causing drug discontinuation

There was no significant difference between the fezolinetant group and the placebo group in terms of drug discontinuation [RR = 1.32, 95% CI (1.00, 1.76), *P* = 0.05] (Figure S6).

#### Headache

There was no significant difference between the fezolinetant group and the placebo group in terms of headache [RR = 1.00, 95% CI (0.81, 1.23), *P* = 1.00] (Figure S7).

#### Arthralgia

Compared to the placebo group, the fezolinetant group had a significantly higher incidence of arthralgia [RR = 2.83, 95% CI (1.02, 7.80), *P* = 0.04] (Figure S8).

#### Nasopharyngitis

There was no significant difference between the fezolinetant group and the placebo group in terms of nasopharyngitis [RR = 0.58, 95% CI (0.26, 1.28), *P* = 0.18] (Figure S9).

#### Nausea

There was no significant difference between the fezolinetant group and the placebo group in terms of nausea [RR = 1.53, 95% CI (0.83, 2.83), *P* = 0.17] (Figure S10).

#### Liver test elevations

There was no significant difference between the fezolinetant group and the placebo group in terms of liver test elevations [RR = 1.16, 95% CI (0.85, 1.58), *P* = 0.36] (Figure S11).

#### Depression

There was no significant difference between the fezolinetant group and the placebo group in terms of depression [RR = 1.00, 95% CI (0.64, 1.56), *P* = 1.00] (Figure S12).

#### Uterine bleeding

There was no significant difference between the fezolinetant group and the placebo group in terms of uterine bleeding [RR = 0.75, 95% CI (0.46, 1.22), *P* = 0.25] (Figure S13).

#### Bone fractures

There was no significant difference between the fezolinetant group and the placebo group in terms of bone fractures [RR = 1.00, 95% CI (0.57, 1.77), *P* = 1.00] (Figure S14).

#### Effect on memory

There was no significant difference between the fezolinetant group and the placebo group in terms of effect on memory [RR = 0.50, 95% CI (0.09, 2.74), *P* = 0.43] (Figure S15).

#### Thrombocytopenia

There was no significant difference between the fezolinetant group and the placebo group in terms of thrombocytopenia [RR = 1.76, 95% CI (0.51, 5.99), *P* = 0.37] (Figure S16).

#### Wakefulness

There was no significant difference between the fezolinetant group and the placebo group in terms of wakefulness [RR = 1.32, 95% CI (0.64, 2.75), *P* = 0.45] (Figure S17).

#### Endometrial hyperplasia or endometrial adenocarcinoma

There was no significant difference between the fezolinetant group and the placebo group in terms of endometrial hyperplasia or endometrial adenocarcinoma [RR = 2.00, 95% CI (0.60, 6.63), *P* = 0.26] (Figure S18).

#### Potential abuse liability

There was no significant difference between the fezolinetant group and the placebo group in terms of potential abuse liability [RR = 1.50, 95% CI (0.42, 5.32), *P* = 0.53] (Figure S19).

### GRADE certainty of evidence

In high-certainty evidence, fezolinetant at 30 mg and 45-mg doses significantly reduced frequency and severity of vasomotor symptoms (VMS) in postmenopausal women. Fezolinetant demonstrated comparable treatment-emergent adverse event (TEAE) rates to placebo, suggesting good tolerability (Additional file (GRADE)).

## Discussion

Fezolinetant has shown significant benefits in managing vasomotor symptoms (VMS) in postmenopausal women, as evidenced by our meta-analysis. Our analysis incorporates five studies with a total of 3295 participants, showcasing a considerable reduction in the frequency and severity of VMS in the fezolinetant group. In addition, our analysis of safety outcomes indicated safety and tolerability of fezolinetant, as a potential therapy for postmenopausal women with VMS.

Fezolinetant dosages of 30 mg and 45 mg once daily were observed to significantly reduce the frequency and severity of VMS. Further, fezolinetant was associated with improvements in MENQOL scores, reflecting an enhanced quality of life, and in PGI-C scores, suggesting patients perceive a positive change in their condition. Additionally, there were numerical improvements in sleep quality for both dosages, as measured by the PROMIS SD SF 8b tool’s total score, with statistical significance reached for the 45-mg dose. This is pertinent since VMS is associated with poor sleep quality, overnight awakenings, and increased daytime drowsiness, and nearly half of postmenopausal women report sleep difficulties. However, the 30 mg dose did not significantly impact sleep quality in this study, likely due to a dosage effect. The analysis of safety outcomes confirmed the safety and tolerability of both the 30 mg and 45 mg doses of fezolinetant, with no significant difference in TEAEs between fezolinetant and placebo.

There are few nonhormonal alternatives for women who cannot or do not want to use HT [26], with only low-dose paroxetine being approved for VMS by the US Food and Drug Administration [27]. The effectiveness of selective serotonin reuptake inhibitors may be compromised in populations with prevalent specific gene polymorphisms, such as the Black population [28]. Off-label use of clonidine, gabapentin, other selective serotonin reuptake inhibitors, serotonin-norepinephrine reuptake inhibitors, and herbal medications are examples of alternative nonhormonal treatments. However, there is either inconsistent information about the efficacy of these medications or they have low efficacy with some tolerability issues [29].

The neurokinin receptor antagonists elinzanetant, pavinetant, and fezolinetant have all been researched for VMS. Comparing fezolinetant to NK1 and NK2 receptor antagonists, fezolinetant is more than 450 times more selective for human NK3R [15]. Phase 3 trials are currently being conducted using elinzanetant, a non-selective NK1R and NK3R antagonist with greater potency at the NK1 receptor [30]. An analysis of the hazards and advantages led to the discontinuation of the possible NK3R antagonist pavinetant [31]. Instead of being a general class effect for NK3R antagonists, observed hepatic adverse effects were hypothesized to be idiosyncratic and connected to the chemical composition of pavinetant [32]. Therefore, fezolinetant remains the best nonhormonal therapeutic option among other nonhormonal and neurokinin receptor antagonist treatments.

Our study’s strengths lie in its comprehensive approach and robust methodology, encompassing a meta-analysis of 3295 participants. The analysis included more than 25 safety and efficacy outcomes with relative subgroup and sensitivity analyses of multiple doses of fezolinetant to comprehensively examine the efficacy and safety of fezolinetant in the treatment of VMS. However, we were limited by a relatively small number of included trials and short trial length.

## Conclusion

Fezolinetant is a safe and effective treatment for postmenopausal females with VMS. Additionally, both doses of 30 mg and 45 mg reduced the frequency and severity of VMS better than the placebo. Furthermore, a 45 mg dose shows a benefit over a 30 mg dose in terms of reducing sleep disruption.

## Supplementary Information

Supplementary Material 1

Supplementary Material 2

## Data Availability

No datasets were generated or analysed during the current study.

## Abbreviations

VMS: Vasomotor symptoms
PGI-C SD: The Patient Global Impression of Change in Sleep Disturbance
PGI-S SD: The Patient Global Impression of Severity in Sleep Disturbance
MENQOL: The Menopause-Specific Quality of Life
PROMIS SD SF 8b: The Patient-Reported Outcomes Measurement Information System Sleep Disturbance—Short Form 8b
TEAEs: Treatment emergent adverse events
AEs: Adverse events
GRADE: Grading of Recommendations, Assessment, Development and Evaluations
