# Supplementary material for: Fezolinetant’s efficacy and safety in treatment of vasomotor symptoms in postmenopausal women: a meta-analysis and GRADE evaluation of randomized controlled trials
Source: Eur J Med Res. 2025 Jan 23;30:52. doi: 10.1186/s40001-025-02279-y (PMC11755967; doi:10.1186/s40001-025-02279-y)
Supplement: Supplementary file 1 — Supplementary Material 1 [file 40001_2025_2279_MOESM1_ESM.docx]

**Supplementary figure legends**

**Figure S1:** Risk of bias summary.

**Figure S2:** A forest plot comparing the prevalence of PGI C-SD in the fezolinetant group versus the placebo group.

**A.** Much better **B.** Moderately better **C.** A little better **D.** No change **E.** A little worse **F.** Moderately worse

**G.** Much worse

**Figure S3:** A forest plot comparing the prevalence of PGI S-SD in the fezolinetant group versus the placebo group.

**A.** No problem **B.** Mild problems **C.** Moderate problems **D.** Severe problems

**Figure S4:** A forest plot comparing the prevalence of drug related AEs in the fezolinetant group versus the placebo group. **A)** Before the sensitivity analysis. **B&C)** After the sensitivity analysis.

**Figure S5:** A forest plot comparing the prevalence of serious TEAEs in the fezolinetant group versus the placebo group.

**Figure S6:** A forest plot comparing the prevalence of TEAEs causing discontinuation in the fezolinetant group versus the placebo group.

**Figure S7:** A forest plot comparing the prevalence of headache in the fezolinetant group versus the placebo group.

**Figure S8:** A forest plot comparing the prevalence of arthralgia in the fezolinetant group versus the placebo group.

**Figure S9:** A forest plot comparing the prevalence of nasopharyngitis in the fezolinetant group versus the placebo group.

**Figure S10:** A forest plot comparing the prevalence of nausea in the fezolinetant group versus the placebo group.

**Figure S11:** A forest plot comparing the prevalence of liver test elevations in the fezolinetant group versus the placebo group.

**Figure S12:** A forest plot comparing the prevalence of depression in the fezolinetant group versus the placebo group.

**Figure S13:** A forest plot comparing the prevalence of uterine bleeding in the fezolinetant group versus the placebo group.

**Figure S14:** A forest plot comparing the prevalence of bone fractures in the fezolinetant group versus the placebo group.

**Figure S15:** A forest plot comparing the prevalence of memory affection in the fezolinetant group versus the placebo group.

**Figure S16:** A forest plot comparing the prevalence of thrombocytopenia in the fezolinetant group versus the placebo group.

**Figure S17:** A forest plot comparing the prevalence of wakefulness in the fezolinetant group versus the placebo group.

**Figure S18:** A forest plot comparing the prevalence of endometrial thickening in the fezolinetant group versus the placebo group.

**Figure S19:** A forest plot comparing the prevalence of potential abuse liability in the fezolinetant group versus the placebo group.


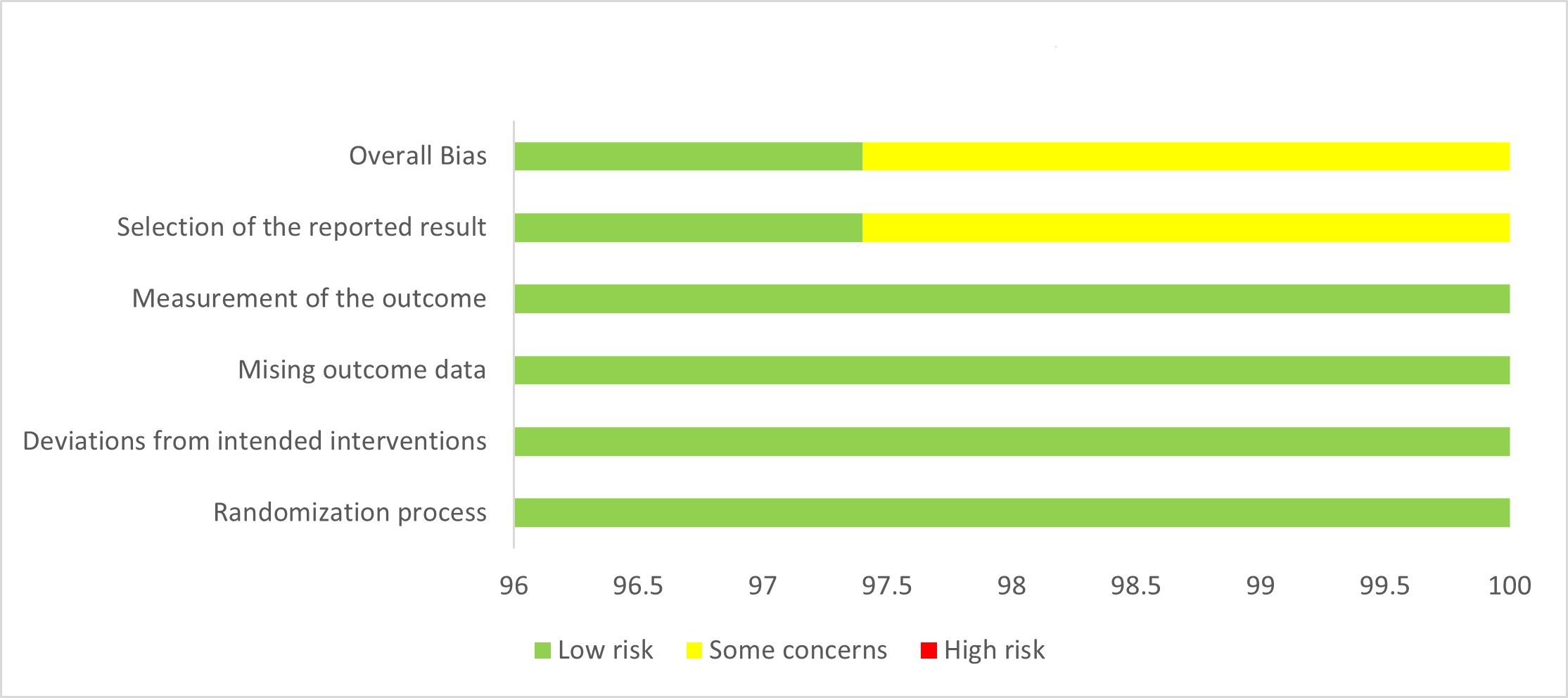


**Figure S1:** Risk of bias summary.


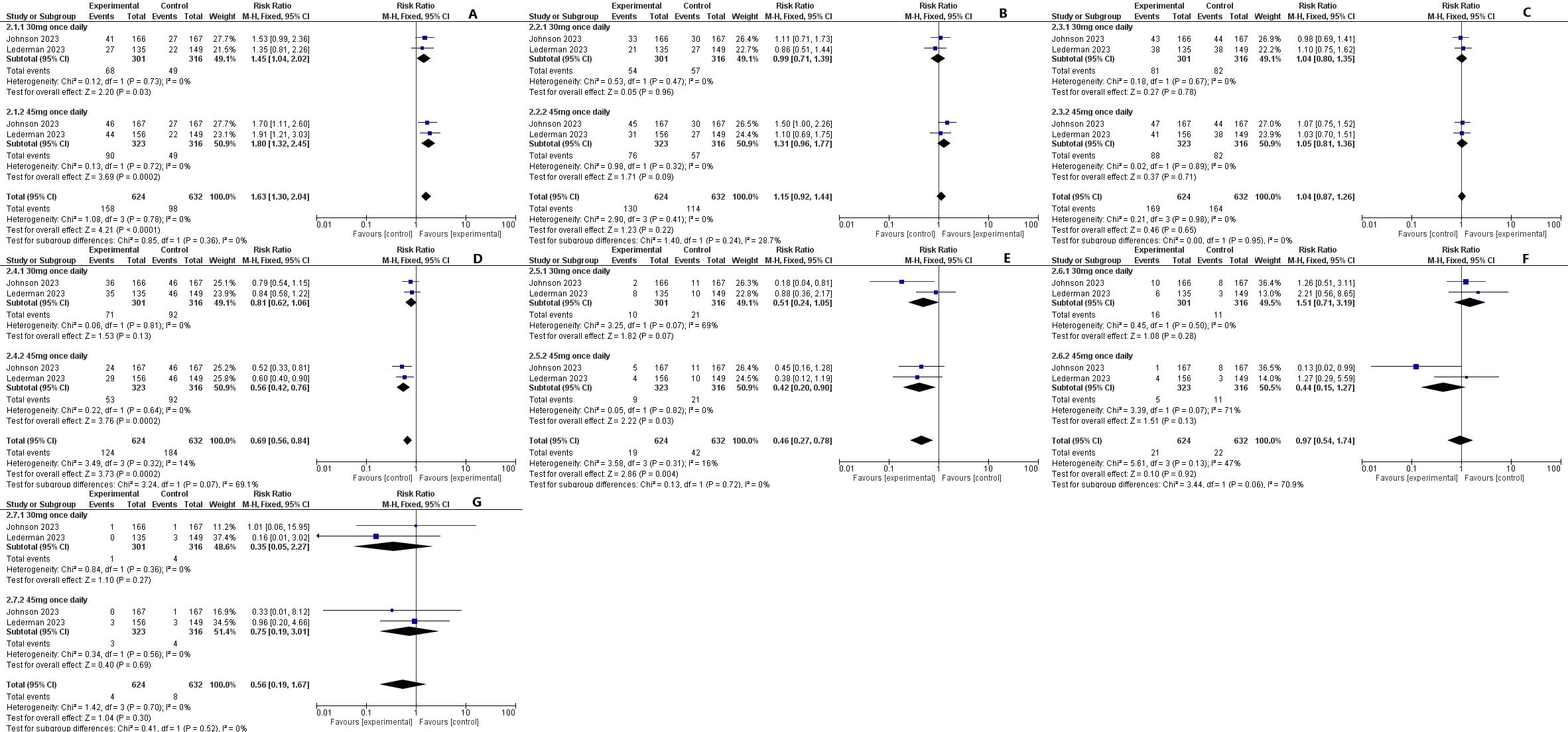


**Figure S2:** A forest plot comparing the prevalence of PGI C-SD in the fezolinetant group versus the placebo group.

**A.** Much better **B.** Moderately better **C.** A little better **D.** No change **E.** A little worse **F.** Moderately worse

**G.** Much worse


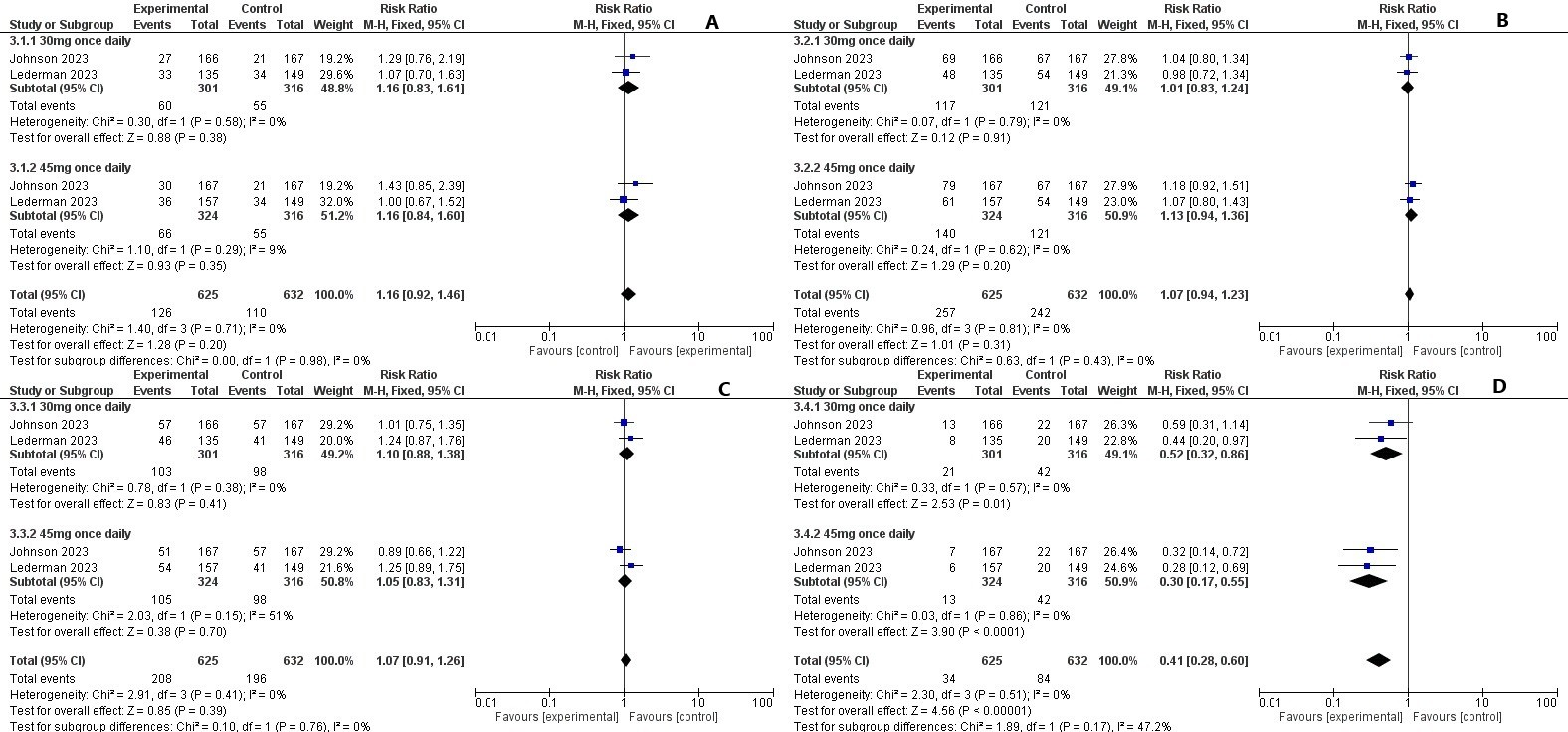


**Figure S3:** A forest plot comparing the prevalence of PGI S-SD in the fezolinetant group versus the placebo group.

**A.** No problem **B.** Mild problems **C.** Moderate problems **D.** Severe problems


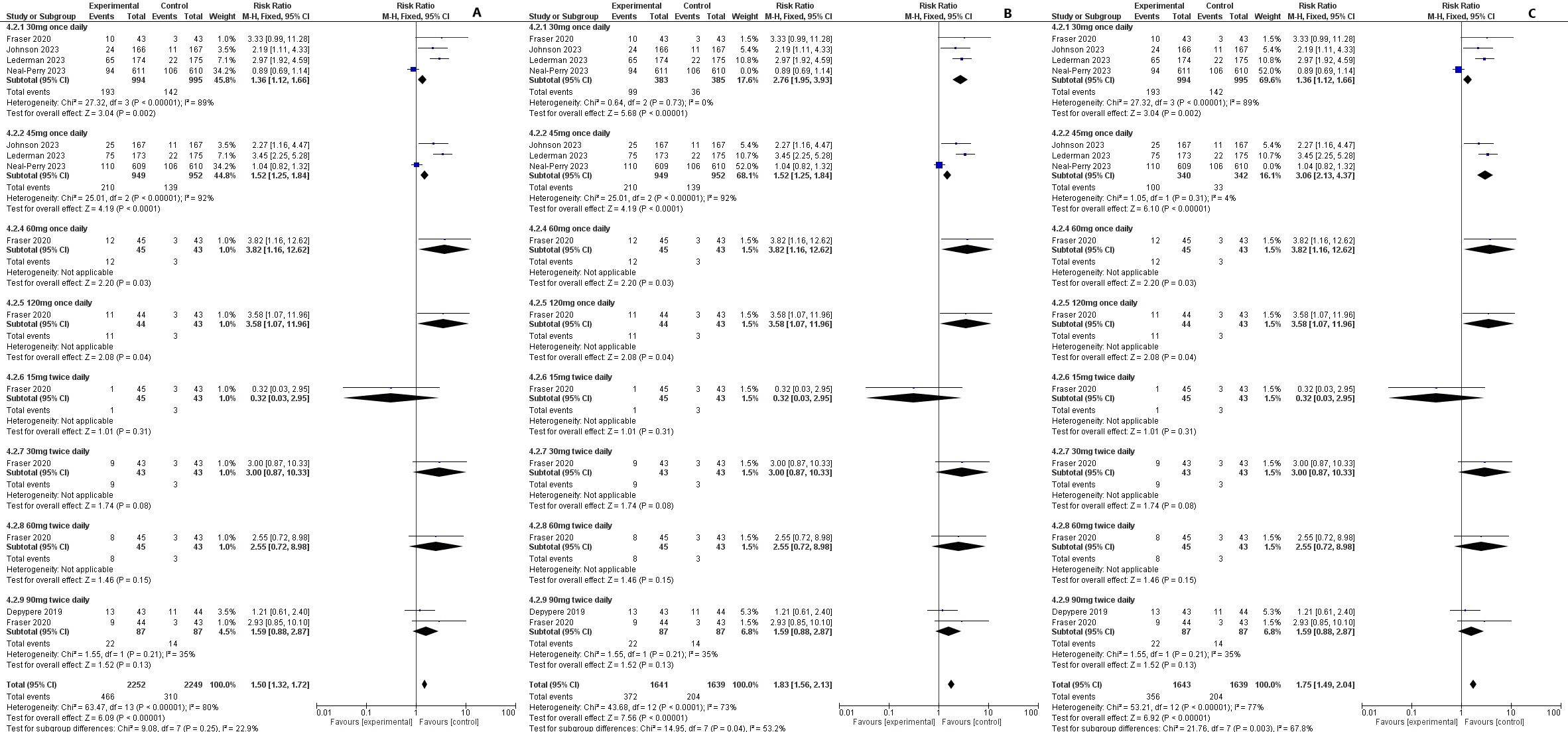


**Figure S4:** A forest plot comparing the prevalence of drug related AEs in the fezolinetant group versus the placebo group. **A)** Before the sensitivity analysis. **B&C)** After the sensitivity analysis.





**Figure S5:** A forest plot comparing the prevalence of serious TEAEs in the fezolinetant group versus the placebo group.





**Figure S6:** A forest plot comparing the prevalence of TEAEs causing discontinuation in the fezolinetant group versus the placebo group.





**Figure S7:** A forest plot comparing the prevalence of headache in the fezolinetant group versus the placebo group.





**Figure S8:** A forest plot comparing the prevalence of arthralgia in the fezolinetant group versus the placebo group.





**Figure S9:** A forest plot comparing the prevalence of nasopharyngitis in the fezolinetant group versus the placebo group.





**Figure S10:** A forest plot comparing the prevalence of nausea in the fezolinetant group versus the placebo group.





**Figure S11:** A forest plot comparing the prevalence of liver test elevations in the fezolinetant group versus the placebo group.





**Figure S12:** A forest plot comparing the prevalence of depression in the fezolinetant group versus the placebo group.





**Figure S13:** A forest plot comparing the prevalence of uterine bleeding in the fezolinetant group versus the placebo group.





**Figure S14:** A forest plot comparing the prevalence of bone fractures in the fezolinetant group versus the placebo group.





**Figure S15:** A forest plot comparing the prevalence of memory affection in the fezolinetant group versus the placebo group.





**Figure S16:** A forest plot comparing the prevalence of thrombocytopenia in the fezolinetant group versus the placebo group.





**Figure S17:** A forest plot comparing the prevalence of wakefulness in the fezolinetant group versus the placebo group.





**Figure S18:** A forest plot comparing the prevalence of endometrial thickening in the fezolinetant group versus the placebo group.





**Figure S19:** A forest plot comparing the prevalence of potential abuse liability in the fezolinetant group versus the placebo group.

**Search strategy**

(Fezolinetant OR ESN364 OR “ neurokinin-3 receptor antagonis”) AND (Menopause OR “Post-menopause” OR “Post menopause” OR “Change of Life, Female”)

**PubMed =** 14

**Scopus =** 23

**WOS =** 17

**Cochrane =** 34

**Total before/after duplication =** 88/52
