# Supplementary material for: Fezolinetant’s efficacy and safety in treatment of vasomotor symptoms in postmenopausal women: a meta-analysis and GRADE evaluation of randomized controlled trials
Source: Eur J Med Res. 2025 Jan 23;30:52. doi: 10.1186/s40001-025-02279-y (PMC11755967; doi:10.1186/s40001-025-02279-y)
Supplement: Supplementary file 2 — Supplementary Material 2 [file 40001_2025_2279_MOESM2_ESM.docx]

| **Summary of findings:** | | | | | | |
| --- | --- | --- | --- | --- | --- | --- |
| **[Fezolinetant] compared to [Placebo] for [Vasomotor symptoms in post-menopausal females]** | | | | | | |
| **Patient or population:** [Vasomotor symptoms in post-menopausal females]  **Setting:** Any  **Intervention:** [Fezolinetant]  **Comparison:** [Placebo] | | | | | | |
| Outcomes | **Anticipated absolute effects^*^** (95% CI) | | Relative effect (95% CI) | № of participants (studies) | Certainty of the evidence (GRADE) | Comments |
|  | **Risk with [Placebo]** | **Risk with [Fezolinetant]** |  |  |  |  |
| Frequency of VMS - 30mg once daily | The mean frequency of VMS - 30mg once daily was **0** | MD **2.13 lower** (2.79 lower to 1.46 lower) | - | 591 (3 RCTs) | ⨁⨁⨁⨁ High | Fezolinetant 30mg once daily results in a reduction in frequency of VMS. |
| Frequency of VMS - 45mg once daily | The mean frequency of VMS - 45mg once daily was **0** | MD **2.62 lower** (3.35 lower to 1.89 lower) | - | 538 (2 RCTs) | ⨁⨁⨁⨁ High | Fezolinetant 45mg once daily results in a reduction in frequency of VMS. |
| Severity of VMS - 30mg once daily | - | SMD **0.26 lower** (0.43 lower to 0.1 lower) | - | 591 (3 RCTs) | ⨁⨁⨁⨁ High | Fezolinetant 30mg once daily results in a reduction in severity of VMS. |
| Severity of VMS - 45mg once daily | - | SMD **0.35 lower** (0.52 lower to 0.18 lower) | - | 538 (2 RCTs) | ⨁⨁⨁⨁ High | Fezolinetant 45mg once daily results in a reduction in severity of VMS. |
| MENQOL - 30mg once daily | The mean MENQOL - 30mg once daily was **0** | MD **0.32 lower** (0.52 lower to 0.13 lower) | - | 564 (2 RCTs) | ⨁⨁⨁⨁ High | Fezolinetant 30mg once daily results in a slight reduction in MENQOL. |
| MENQOL - 45mg once daily | The mean MENQOL - 45mg once daily was **0** | MD **0.49 lower** (0.67 lower to 0.3 lower) | - | 593 (2 RCTs) | ⨁⨁⨁⨁ High | Fezolinetant 45mg once daily results in a slight reduction in MENQOL. |
| TEAEs - 30mg once daily | 547 per 1,000 | **574 per 1,000** (530 to 618) | **RR 1.05** (0.97 to 1.13) | 1989 (4 RCTs) | ⨁⨁⨁⨁ High | Fezolinetant 30mg once daily results in little to no difference in TEAEs. |
| TEAEs - 45mg once daily | 549 per 1,000 | **549 per 1,000** (511 to 599) | **RR 1.00** (0.93 to 1.09) | 1901 (3 RCTs) | ⨁⨁⨁⨁ High | Fezolinetant 45mg once daily results in little to no difference in TEAEs. |
| ***The risk in the intervention group** (and its 95% confidence interval) is based on the assumed risk in the comparison group and the **relative effect** of the intervention (and its 95% CI).  **CI:** confidence interval; **MD:** mean difference; **RR:** risk ratio; **SMD:** standardised mean difference; **VMS:** vasomotor symptoms; **MENQOL:** Menopause-Specific Quality of Life; **TEAEs: treatment emergent adverse events.** | | | | | | |
| **GRADE Working Group grades of evidence** **High certainty:** we are very confident that the true effect lies close to that of the estimate of the effect. **Moderate certainty:** we are moderately confident in the effect estimate: the true effect is likely to be close to the estimate of the effect, but there is a possibility that it is substantially different. **Low certainty:** our confidence in the effect estimate is limited: the true effect may be substantially different from the estimate of the effect. **Very low certainty:** we have very little confidence in the effect estimate: the true effect is likely to be substantially different from the estimate of effect. | | | | | | |
